# Exposure to environmental phenols and parabens, and relation to body mass index, eczema and respiratory outcomes in the Norwegian RHINESSA study

**DOI:** 10.1186/s12940-021-00767-2

**Published:** 2021-07-13

**Authors:** Hilde Kristin Vindenes, Cecilie Svanes, Stein Håkon Låstad Lygre, Francisco Gomez Real, Tamar Ringel-Kulka, Randi Jacobsen Bertelsen

**Affiliations:** 1 Department of Occupational Medicine, Haukeland University Hospital, Bergen, Norway; 2 Department of Clinical Science, University of Bergen, Bergen, Norway; 3 Centre for International Health, University of Bergen, Bergen, Norway; 4 Department of Maternal and Child Health, Gillings School of Global Public Health, University of North Carolina, Chapel Hill, NC USA; 5 Oral Health Center of Expertise, Western Norway, Bergen, Norway

**Keywords:** Chemicals, Phenols, BMI, Exposure, Allergy, Personal care products, RHINESSA

## Abstract

**Background:**

Many phenols and parabens are applied in cosmetics, pharmaceuticals and food, to prevent growth of bacteria and fungi. Whether these chemicals affect inflammatory diseases like allergies and overweight is largely unexplored. We aimed to assess the associations of use of personal care products with urine biomarkers levels of phenols and paraben exposure, and whether urine levels (reflecting body burden of this chemical exposures) are associated with eczema, rhinitis, asthma, specific IgE and body mass index.

**Methods:**

Demographics, clinical variables, and self-report of personal care products use along with urine samples were collected concurrently from 496 adults (48% females, median age: 28 years) and 90 adolescents (10–17 years of age) from the RHINESSA study in Bergen, Norway. Urine biomarkers of triclosan (TCS), triclocarban (TCC), parabens and benzophenone-3, bisphenols and dichlorophenols (DCP) were quantified by mass spectrometry.

**Results:**

Detection of the urine biomarkers varied according to chemical type and demographics. TCC was detected in 5% of adults and in 45% of adolescents, while propyl (PPB) and methyl (MPB) parabens were detected in 95% of adults and in 94% (PPB) and 99% (MPB) of adolescents. Women had higher median urine concentrations of phenolic chemicals and reported a higher frequency of use of personal care products than men. Urine concentration of MPB increased in a dose-dependent manner with increased frequency of use of several cosmetic products. Overall, urinary biomarker levels of parabens were lower in those with current eczema. The biomarker concentrations of bisphenol S was higher in participants with positive specific IgE and females with current asthma, but did not differ by eczema or rhinitis status. MPB, ethylparaben (EPB), 2,4-DCP and TCS were inversely related to BMI in adults; interaction by gender were not significant.

**Conclusions:**

Reported frequency of use of personal care products correlated very well with urine biomarker levels of paraben and phenols. Several chemicals were inversley related to BMI, and lower levels of parabens was observed for participants with current eczema. There is a need for further studies of health effects of chemicals from personal care products, in particular in longitudinally designed studies.

**Supplementary Information:**

The online version contains supplementary material available at 10.1186/s12940-021-00767-2.

## Background

The use of personal care and beauty products has increased a lot in the last decades. These products are in daily contact with the skin, but there is limited knowledge on the relationship between pattern of use of different products, absorption and associations with health outcomes. A number of the chemicals may affect the epithelial barrier. Thereby inflammatory responses may be induced and influence health outcomes such as allergy and obesity, both linked to inflammation [1]. Synthetic phenolic compounds like parabens and triclosan (TCS) are broad-spectrum antimicrobial chemicals that are used as preservatives in cosmetics, pharmaceuticals, food, food-wrapping and kitchen utensils [2, 3]. The chemicals are added to the products to prevent growth of bacteria and fungi [4]. As with triclosan, parabens are commonly found in personal care products [5], but methyl (MPB) and propyl (PPB) parabens are also used in food [6, 7]. TCS is now being phased out in many countries and sometimes replaced by triclocarban (TCC) [8, 9]. Benzophenone-3 (BP3), a sunscreen agent, is another chemical that is used in increasing amount in personal care products [10, 11], mainly as an UV filter, but also in other materials to prolong product durability. 2,5-dichlorophenol (2,5-DCP) is used in moth balls as deodorizer for room and toilets [12], whereas the sister-compound, 2,4-DCP, a photo degradation product of triclosan [12], is used as a pesticide and a fungicide for instance as preservative in citrus fruits and vegetables. Bisphenol analogues are present in a variety of consumer products and foods [13]. They are found in different cosmetics products, mainly due to contamination of bisphenols released from bisphenol-contaning cosmetics containers [14]. The main routes of exposure in humans to non-persistent phenolic compounds like parabens, TCS and BP3 are through the mouth or skin, with the most important sources being food and personal care products [3, 10, 15, 16]. The chemicals are excreted from the body mainly through urine, and the biomarkers that are measurable in urine represent all routes of exposure; oral, inhaled and dermal [17–19]. To a lesser degree, these phenolic compounds are detectable in other matrices such as breast milk and serum [17]. These compounds, which to date have been considered to be non-persistent, may deposit in human adipose tissue [20, 21]. It has also been speculated whether chronic obesity may induce low grade inflammation [22], and thereby trigger immunological changes associated with a cytokine release pattern that increases the risk of allergy [23]*.* Vieira et al. found that the frequency of IgE-positivity in response to a balanced mixture of common aeroallergens was three-fold greater in obese than non-obese women [24] and a meta-analysis showed that overweight/obesity was associated with an increased prevalence of AD, particularly in children and adults in North America and Asia [25]. In previous studies urinary levels of TCS and parabens have been associated with aeroallergen and food sensitization [26, 27]. Prenatal 2,5-dichlorophenol (2,5-DCP) urine concentration has been associated with eczema and respiratory outcomes, but among boys only [28]. Others have suggested a potential effect of phenols and parabens on risk of asthma and allergy in children [29]. A few previous publications have reported on phenolic compound exposure from Norwegian population [30, 31], but associations with BMI measures and allergic outcomes have not been explored.

In this study, we aim to describe urine biomarkers of exposure to common environmental and cosmetic chemicals in a Norwegian population and their association with self-reported personal care products use. We want to examine chemical exposure in both adolescents and adults as potential differences in exposures levels and sources may be present. Secondly, we aim to assess whether exposure to these phenols is associated with eczema, rhinitis, asthma, specific IgE, and BMI.

## Methods

### Study population and sample collection

The study population included males and females in the age-range from 10 to 47 years of age investigated as part of the RHINESSA generation study [32] in Bergen, Norway in 2014–2015 (www.rhinessa.net). The RHINESSA study population consists of offspring of participants in the population-based multi-centre European Community Respiratory Health Survey (ECRHS, www.ecrhs.org) and daughter study Respiratory Health in Northern Europe (RHINE, www.rhine.nu). Offspring in all ages were identified from population registries and invited to the RHINESSA study. The participants completed questionnaires, interviews and clinical examination. Information on symptoms and history of diseases, smoking habits and the home environment was collected. They also answered a separate questionnaire about frequency of use of 15 personal care products. The study protocols were harmonized with the ECRHS and RHINE studies, and the questionnaires are available at www.rhinessa.net.

The clinical examination included weight, height, hip and waist circumference measurements in addition to blood and urine sampling. The time of day for collection of samples as well as information of whether the participants had been fasting on the day of sample collection were registrated.

Approval was obtained from the Regional Committee for Medical and Health Research Ethics in Western Norway (approval number 2012/1077). All participants provided written informed consent.

### Quantification of urine biomarkers of chemical exposure

Urine specific gravity (SG) was measured by a handheld Atago refractometer PAL 10-S (ATAGO Co., Ltd, Bellevue, WA, USA) before freezing the urine in polypropylene tubes (-80 °C) without preservatives. The urine collection tubes contain no phenolic compounds and thus should not interfere with the phenolic quantification. The urine biomarkers analysis was done at CDC (Atlanta, GA, USA). The total (free + conjugated) concentrations of 12 phenol- and phenol-like compounds (parabens, TCS, TCC, BP3, 2,4-DCP, 2,5-DCP, bisphenol A (BPA), bisphenol S (BPS), bisphenol F (BPF)) was measured using a modification of the automated online solid-phase extraction high-performance liquid chromatography-tandem mass spectrometry method reported in Ye et al. [33]. This method has good reproducibility and accuracy and permits quick and accurate analysis of a large number of samples as demonstrated in studies assessing the prevalence of human exposure to parabens [19]. The limits of detection (LOD) for the various chemicals as well as the percentages of samples above LOD are provided in Table 2. The LOD values were determined during the laboratory procedure. Urinary concentrations of parabens, bisphenols, dichlorophenols, TCS, TCC and BP3 were standardized to urine specific gravity (SG) to avoid the influence of urine volume fluctuation, using the following formula: Ps = P [1.019 – 1)/ (SGi – 1)] where Ps is the SG-standardized concentrations, P is the measured concentration, 1.019 is the median urinary specific gravity in this population, and SGi is the urine specific gravity of the individual sample. We created molar sum of parabens by summing the individual paraben concentrations in micrograms per liter divided by the molecular weight of the parabens. Time of day of urine sampling was devided in 4 time categories.

### Definition of eczema, rhinitis, asthma and spesific IgE

Current eczema was defined as having itchy rash coming and going for at least 6 months and having had the itchy rash in the last 12 months. Current asthma was defined as asthma attack last 12 months and/or use of current asthma medication. Current rhinitits was defined as having a positive response to question on having any nasal allergies including hay fever and having sneezing/runny blocked nose without a cold the last 12 months. These outcomes are defined from questionnaires and interviews based on the European Community Respiratory Health Study (www.ecrhs.org), which are used in studies across the world and are considered international standard [34–36].

Total and specific IgE were measured according to standardized laboratory methods at Haukeland University Hospital in Bergen, Norway. IgE positivity was defined by IgE ≥ 0.35 kU/L to at least 1 of 5 allergens tested (cat, timothy grass, Cladosporium, birch, and house dust mite) measured by ImmunoCAP (Phadia AB, Uppsala, Sweden). The laboratory did not provide IgE values below 0.35 kU/L.

### Definition of body mass index

BMI was calculated from measured height and weight (weight (kg)/height squared (m^2^)) and used to classify adults as underweight (BMI < 18.5 kg/m^2^), normal weight (BMI ≥ 18.5, < 25.0 kg/m^2^), overweight (BMI ≥ 25.0, < 30.0 kg/m^2^), or obese (BMI ≥ 30.0 kg/m^2^). BMI categorization of children from 10 to 18 years was done according to international BMI definitions for normal weight, overweight or obese, using BMI cutoffs recommended by the Childhood Obesity Working Group of the International Obesity Taskforce [37].

### Statistical methods

In the statistical analyses applying urine biomarker concentrations, we used machinery readings when these were available for values below the detection level. It is recommended that statistical processing of datasets containing low concentrations of analyte should be undertaken on data containing machine read values when these are available, rather than censoring values below the limit of detection [38], to avoid negative bias at low concentrations. For values without any machine read values, we used values of LOD/√2. Specific gravity-adjusted concentration measures were used in all statistical analyses as they are less affected by body mass index (BMI), muscle mass, age, gender, diet, activity and season compared to creatinine-adjusted concentrations [39]. Spearman correlation was used to estimate correlation coefficient, r, between levels of chemicals.

Chemical biomarkers were measured in 499 adults and 91 adolescents. Among these, metadata (including information on gender) was missing for three adult participants and one adolescent, and these are therefore excluded from analyses which takes metadata into account. Possible impact on eczema, rhinitis, asthma and total specific IgE from levels of chemicals in urine samples were assessed with adjustment for possible confounding from BMI, IgE, age, smoking and time of day of sampling. The exposure-outcome associations were restricted to adult participants (≥ 18 years) due to low number of adolescents in the study population. Smoking was included as this is likely to have immunomodulatory effects; and time of sampling as this may influence urine concentration of chemicals. Possible differences in participants characteristics between genders were tested by t-test (continuous variables) and Pearson's chi square test (categorical variables) (Table 1). Pearson's chi square test was used to assess possible differences in frequency of use of personal care products between men and women (Table 3).

**Table 1:** Participant characteristics by gender for adults (18 +) and adolescents [10–17 years]

	**Adults (≥ 18 years)**			**Adolescents (10–17 years)**		
	**All (*n* = 496)**	**Men (*n* = 258)**	**Women (*n* = 238)**	**All (*n* = 90)**	**Boys (*n* = 46)**	**Girls (*n* = 44)**
**Age** [median (min–max)]	27.4 (18.1–47.5)	28.3 (18.1- 47.5)	26.4 (18.1- 45.2) *	14.9 (9.8–17.9)	15.3 (11.0–17.9)	14.6 (9.7–17.8)
**Education (%)**						
Primary school	2.1	3.3	0.9	60.7	56.5	65.1
Secondary school	40.6	45.0	36.1	39.3	43.5	34.9
University/college	57.3	51.7	63.0*			
**Smoking(cigarettes) (%)**						
Never	64.4	62.3	66.5	95.4	93.3	97.6
Previous	24.2	23.7	24.8	2.3	2.2	2.4
Current	11.4	14.0	8.7	2.3	4.4	-
**Fasting(% yes)**	41.6	41.2	42.0	28.1	26.7	29.6
**Time of urine collection (%)**						
Before 10 AM	18.4	16.8	20.2	13.3	10.9	15.9
10 AM-12 PM	39.3	39.1	39.5	31.1	34.8	27.3
12–2 PM	37.3	39.9	34.4	25.6	19.6	31.8
After 2 PM	5.0	4.3	5.9	30.0	34.8	25.0
**Current eczema (% yes)**	11.3	9.5	13.3	12.2	10.9	13.6
**Current rhinitis (% yes)**	44.1	43.6	44.7	31.1	39.1	22.7
**Current asthma (% yes)**	6.75	4.7	8.9	11.6	9.3	13.9
**Spesific IgE (% yes)**	43.5	50.5	35.8**	56.0	65.9	44.1
**BMI-group (kg/m**^**2**^**)(%)**						
< 18.5	1.8	1.2	2.5	7.0	4.5	9.5
≥ 18.5–25	56.5	44.2	69.8	75.6	75.0	76.2
≥ 25–30	30.4	41.4	18.5	15.1	18.2	11.9
≥ 30	11.3	13.2	9.2**	2.3	2.3	2.4

^*^P-value < 0.05 and **P-value < 0.01 for difference between gender categories (Pearson’s chi square for categorical variable, t-test for continuous variables). Information on gender was missing for 4 participants (3 adults and 1 adolescent not included in the table) who had provided urine samples. BMI categories for adolescents: international BMI definitions for normal weight, overweight or obese. IgE positivity defined by IgE ≥ 0.35 kU/L to at least 1 of 5 allergens tested (cat, timothy grass, Cladosporium, birch, and house dust mite). Observation missing: Education: 28 (adults), 1 (adolescent); smoking: 30 (adults), 3 (adolescents); Fasting: 1 (adult); Current eczema: 18 (adults), Current asthma: 7 (adults), 4 (adolescents); Current rhinitits: 18 (adults); specific IgE: 18 (adults), 15 (adolescents), BMI;3 (adolescent); time of urine collections: no missing

The main variables of interest were exposures to the chemicals PPB, MPB, EPB, butyl paraben (BPB), TCS, TCC, BP3, bisphenol A; F; S, 2,4-DCP and 2,5-DCP. Multiple linear regression models adjusted for BMI, specific IgE, smoking, age and time of day when urine was collected with clustering for siblings, were applied to model possible association between specific gravity standardized urine biomarker concentrations of these phenols with BMI and current eczema, rhinitis, asthma and specific IgE. The chemicals for which more than 50% of the samples had undetectable exposure levels, were treated as dichotomous variables (below LOD vs above LOD), and modelled with logistic regression models. Possible effect modification on BMI, eczema, rhinitis, asthma and spesific IgE from gender were investigated by also including appropriate interaction terms in the models.

## Results

The study population included 496 adults (47.8% women) and 90 adolescents (48.8% girls) from the RHINESSA study center in Bergen, Norway. The study included all participants with available urine samples until the urine samples were shipped to the laboratory for analyses, thus, there were no sub-selection of study participants for this particular study. The women were younger than the men (median age: 26 vs 28 years, respectively) and had also higher educational level, were less often obese or overweight and less likely to be current smokers as compared to the male participants (Table 1). There was no gender difference with regard to whether they had been fasting before sample collection or for time of day of urine collection (Table 1).

**Fig. 1:**
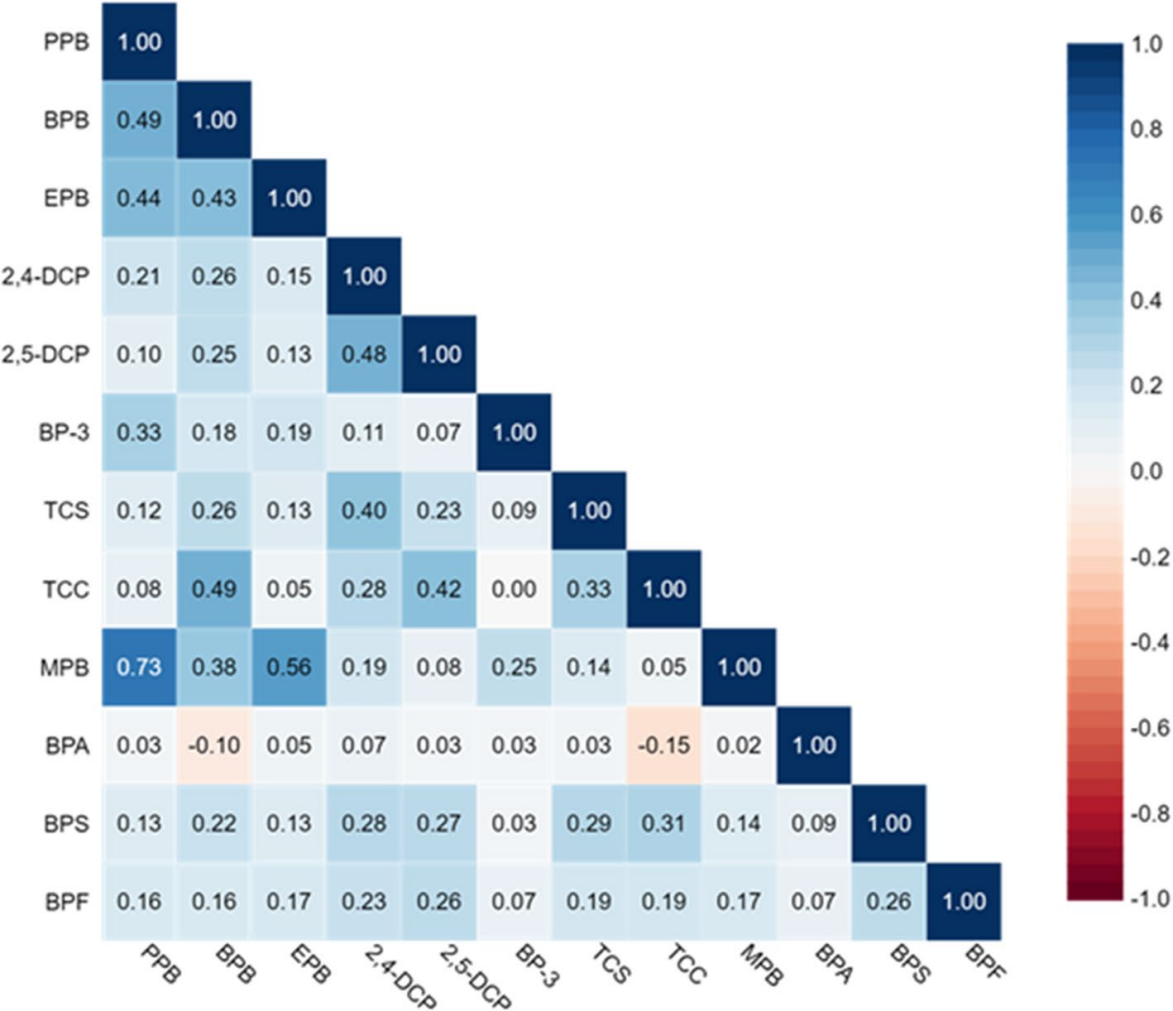
Spearman Correlation matrix for chemicals by urine measurements in adults

**Fig. 2:**
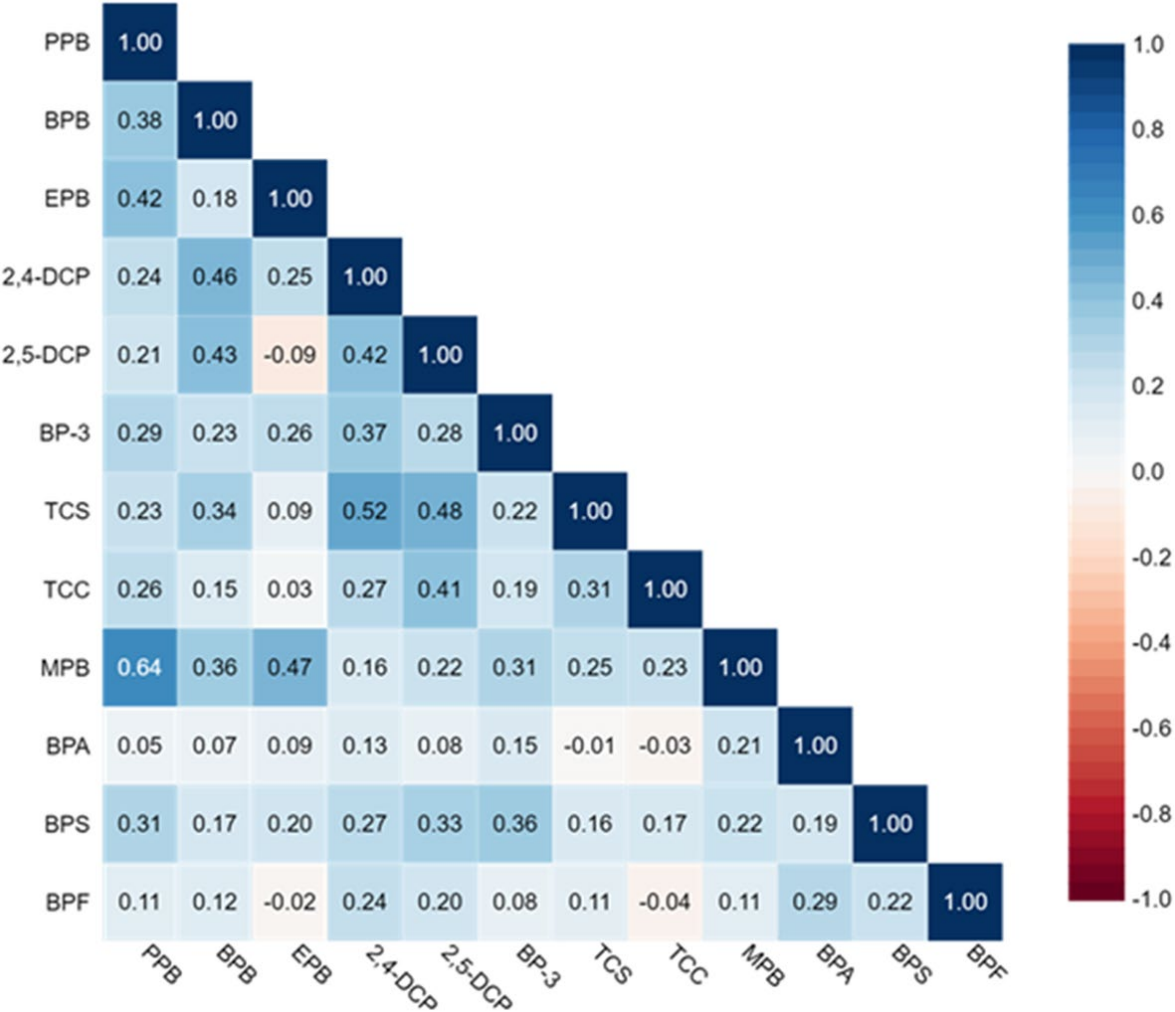
Spearman Correlation matrix for chemicals by urine measurements in adolescents

There was a large variability in detectable levels of the measured urine chemicals, with the highest level of detection for MPB (95%), PPB (95%), BPA (95%) and BP3 (86%) in adults (Table 2), and with similar observations for adolescents (Table 2). Compared to adults, participants under the age of 18 had lower median concentrations of all phenols. Triclocarban was above the LOD in only 5% of the samples and TCS in 27% of the samples for adults compared to 45% and 19% for adolescents, respectively. TCC showed the highest concentration for the 12–18 year age group (Supplementary Figure S1). Bisphenols were also more frequently detected in adolescents than in adults (Table 2), but with higher urine concentrations among exposed adults than among the exposed adolescents. There was a strong correlation between MPB and PPB (*r* = 0.73 (adults, Fig. 1) and r = 0.64 (adolescents, Fig. 2), but less so among the other parabens (r between 0.6 and 0.4), whereas the correlation was weak between the other chemicals. Furthermore, men and women differed in their exposure to chemicals; in terms of overall concentrations, but also by having different exposure-trajectory by age (Supplementary Figure S1). Women had a higher median urine concentration of phenolic compounds compared to men, except for BPA (Supplementary Table S1). The median urine concentration of MBP were almost 10 times higher in women than in men (Supplementary Table S1).

**Table 2:** Distribution of SG-corrected urinary phenols and triclocarban concentrations (ng/mL) among 499 adult and 91 adolescent RHINESSA participants

Analyte (Long name)	Analyte	LOD	Group	% ≥ LOD	5th	25th	50th	75th	95th	Max
Methylparaben	MPB	1.0	Adult	95.2	1.30	4.60	19.3	95.6	386	3727
			Adolescents	98.9	1.52	2.69	5.63	23.1	130	553
Ethylparaben	EPB	1.0	Adult	61.9	< LOD	< LOD	1.71	5.30	54.0	2957
			Adolescents	39.5	< LOD	< LOD	< LOD	1.28	8.06	412
Propylparaben	PPB	0.1	Adult	95.2	0.12	0.42	1.72	11.2	112	781
			Adolescents	94.5	< LOD	0.26	0.78	3.45	14.9	187
Butylparaben	BPB	0.1	Adult	38.1	< LOD	< LOD	0.11	0.31	6.53	137
			Adolescents	24.2	< LOD	< LOD	< LOD	0.18	1.87	23.1
Bisphenol A	BPA	0.2	Adult	95.4	0.43	0.87	1.33	2.43	5.03	21.9
			Adolescents	95.6	0.33	0.62	0.97	1.43	3.54	7.1
Bisphenol F	BPF	0.2	Adult	50.1	< LOD	< LOD	0.28	0.50	2.35	80.6
			Adolescents	69.2	< LOD	< LOD	0.27	0.44	1.57	4.4
Bisphenol S	BPS	0.1	Adult	62.1	< LOD	0.10	0.15	0.23	0.52	8.4
			Adolescents	71.4	< LOD	< LOD	0.14	0.26	0.52	7.2
Benzophenone-3	BP-3	0.4	Adult	86.4	< LOD	2.29	7.90	28.7	396	6041
			Adolescents	94.5	0.41	1.36	4.14	12.8	130	1893
2,5-diclorophenol	2,5-DCP	0.1	Adult	38.3	< LOD	< LOD	0.12	0.18	0.62	23.5
			Adolescents	28.6	< LOD	< LOD	< LOD	0.14	0.57	5.7
2,4-diclorophenol	2,4-DCP	0.1	Adult	65.7	< LOD	0.11	0.16	0.22	0.63	7.4
			Adolescents	62.6	< LOD	< LOD	0.12	0.20	0.45	2.7
Triclosan	TCS	1.7	Adult	26.8	< LOD	< LOD	< LOD	2.35	48.5	861
			Adolescents	18.7	< LOD	< LOD	< LOD	2.20	38.1	451
Triclocarban	TCC	0.1	Adult	5.4	< LOD	< LOD	< LOD	0.12	0.28	0.9
			Adolescents	45.1	< LOD	< LOD	< LOD	0.20	0.45	1.3

LOD: Limit of detection

**Fig. 3:**
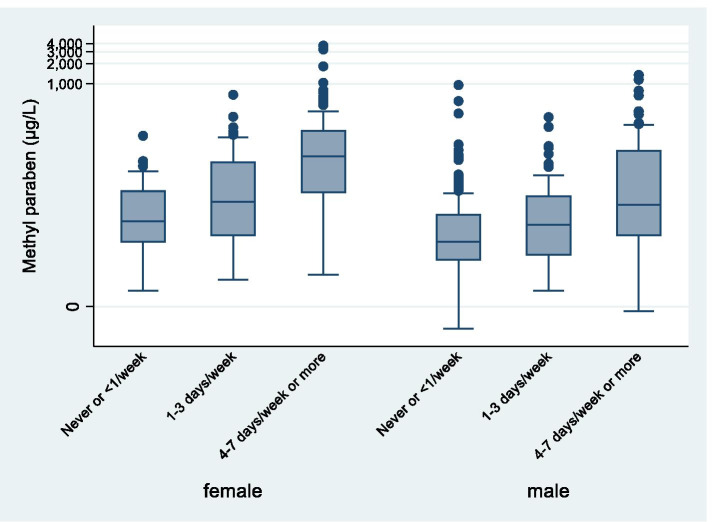
Methylparaben by moisturizer use, adult participants

Females reported more frequent use of the majority of the personal care products, except for hair-styling products, deospray and shaving products, which males used more frequently than females (Table 3). Urinary concentrations of parabens increased in a dose–response manner with frequency of use of many of the personal care products especially for products applied over a wider skin surface and for “leave on” products, for instance moisturizer and lotions (results for MPB reported in Supplementary Table S2). This was true for participants both over and under 18 years. Figure 3 shows that the urine concentration of MPB increased with increasing frequency of reported use of moisturizer in both women and men, but that the urine concentrations of MPB were overall higher in women than in men.

**Table 3:** Reported frequency of use of personal care products by men and women stratified by adults and adolescents

		**Adults (≥ 18 years)**			**Adolescents (10–17 years)**		
		Frequency of use (%)			Frequency of use (%)		
Products		**Never or < 1/week**	**1–3 days/week**	**4–7 days/week or > once daily**	**Never or < 1/week**	**1–3 days/week**	**4–7 days/week or > once daily**
Perfume spray	Women	23.7	17.4	58.9	29.6	29.6	40.9
	Men	42.6	19.0	38.4 ***	48.9	17.8	33.3
Perfume (not spray)	Women	91.1	2.2	6.7	97.7	2.3	0
	Men	93.0	4.7	2.3	91.1	2.2	6.7
Deo-spray	Women	97.8	0.4	1.8	97.7	-	2.3
	Men	87.0	4.7	8.3***	82.2	6.7	11.1*
Deostick	Women	2.1	3.9	94.0	11.4	13.6	75.0
	Men	9.7	4.7	85.6**	17.8	13.3	68.9
Moisturizing cream	Women	8.5	17.8	73.7	34.1	22.7	43.2
	Men	56.4	21.0	22.6***	73.3	11.1	15.6**
Lotions	Women	26.3	31.4	42.4	52.3	36.4	11.4
	Men	90.3	5.8	3.9***	86.7	6.7	6.7**
Cleansing cream	Women	35.3	14.9	49.8	65.9	18.2	15.9
	Men	93.7	4.3	2.0***	86.7	6.7	6.7
Make-up	Women	7.7	14.9	77.4	25.0	11.4	63.6
	Men	100	-	-***	100	-	-***
Nail-care	Women	72.0	20.8	7.2	67.4	25.6	6.9
	Men	100	-	-***	100	-	-***
Shaving-products	Women	84.7	13.6	1.7	84.1	15.9	-
	Men	51.2	42.8	6.0 ***	93.3	4.4	2.2
After-shave	Women	98.2	0.9	0.9	100	-	-
	Men	83.3	14.3	2.4***	100	-	-
Hairspray	Women	69.4	18.7	11.9	93.2	6.8	-
	Men	95.3	3.9	0.8***	97.8	2.2	-
Hair-styling products	Women	65.3	18.6	16.1	93.2	4.5	2.3
	Men	41.4	17.1	41.7 ***	22.2	20.0	57.8***
Hair-color^a^	Women	57.1	31.5	11.6	86.4	13.6	-
	Men	99.2	0.8	-***	100	-	-**
Hair-bleach^a^	Women	69.8	25.0	5.2	88.6	9.1	2.3
	Men	99.6	0.4	-***	100	-	-

^*^P-value ≤ 0.05; **P-value ≤ 0.01; ***P-value ≤ 0.001 for difference in frequencies of use between genders (Pearson’s chi square test) ^a^Frequency of use categorie: Never or less than 1 time/year, 1–6 times/year, 6–12 times/year or more than once/month)

In the exposure-outcome models (adult participants only), urine biomarker concentration of parabens (parabensum, PBP, MPB and BBP) was lower for participants with eczema (Table 4). Furthermore, butylparaben was also inversely associated with current rhinitis (p = 0.05) (Table 5). For BPA, a significant interaction (p = 0.01) was found for sex and rhinitis, and stratified by sex, we found that BPA concentration was decreased with rhinitis for men (beta-coefficient -0.24 (95% CI: -0.45, -0.04)), and not for women (beta-coefficient 0.13 (95% CI: -0.06, 0.32)). For asthma there was a significant interaction for sex and BPS (Table 6), and when stratified by sex, we found that the concentration was increased with current asthma for women (beta-coefficient 0.29 (95% CI: 0.02, 0.57)), but not in men (beta-coefficient -0.25 (95% CI: -0.51, 0.01)). For parabensum and methylparaben and current asthma, there was a significant interaction with sex, however, no significant result when stratified by gender.

**Table 4:** Associations between urine biomarkers of chemical exposure and current eczema in adult participants

	**Sex: male vs female**			**Current eczema**			**Interaction**^**c**^
	**∆/OR**	**CI**	**p**	**∆/OR**	**CI**	**p**	**p**
Sum of paraben^a^	-0.71	(-0.85,-0.56)	< 0.001	**-0.24**	**(-0.43,0.05)**	**0.01**	0.4
Methylparaben^a^	-1.76	(-2.10,-1.42)	< 0.001	**-0.62**	**(-1.06,0.18)**	**0.006**	0. 7
Butylparaben^b^	0.12	(0.07,0.19)	< 0.001	**0.53**	**(0.28,1.01)**	**0.05**	NT
Propylparaben^a^	-2.24	(-2.61,-1.87)	< 0.001	**-0.70**	**(-1.22,0.18)**	**0.009**	0.8
Ethylparaben^a^	-1.11	(-1.42,-0.80)	< 0.001	-0.33	(-0.76,0.09)	0.12	0.09
Benzophenone-3^a^	-1.52	(-1.94,-1.10)	< 0.001	-0.11	(-0.78,0.55)	0.7	0.9
Bisphenol S^a^	-0.06	(-0.19,0.07)	0.4	-0.10	(-0.28,0.08)	0.2	1.0
Bisphenol F^a^	-0–14	(-0.35,0.06)	0.2	-0.16	(-0.48,0.16)	0.3	0.7
Bisphenol A^a^	0.15	(0.00,0.31)	0.05	-0.00	(-0.25,0.24)	1.0	0. 2
2,4- DCP^a^	-0.06	(-0.19,0.08)	0.42	-0.06	(-0.27,0.15)	0.4	0.2
2,5 –DCP^*b*^	1.79	(1.16,2.75)	0.01	0.88	(0.49,2.1.60)	0.7	0.6
Triclosan^b^	0.65	(0.40,1.06)	0.08	0.75	(0.36,1.59)	0.5	0.4
Triclocarban^b^	0.58	(0.23,1.46)	0.3	1.74	(0.54,5.67)	0.2	0.7

^a^Multiple linear or ^b^logistic regression including sex and current eczema adjusted for BMI, IgE, age, smoking, and time of day of urine sampling; ^c^ Interaction term between sex and current eczema. NT (not testable); no men with both current eczema and butylparaben in urine

**Tabel 5:** Associations between urine biomarkers of chemical exposure and current rhinitis in adult participants

	**Sex: male vs female**			**Current rhinitis**			**Interaction**^**c**^
	**∆/OR**	**CI**	**p**	**∆/OR**	**CI**	**p**	**p**
Sum of paraben^a^	-0.69	(-0.84,-0.54)	< 0.001	-0.14	(-0.29,0.01)	0.06	0.3
Methylparaben^a^	-1.72	(-2.06–1.38)	< 0.001	-0.27	(-0.59,0.06)	0.1	0.3
Butylparaben^b^	0.12	(0.07,0.19)	< 0.001	0.61	**(0.37,1.00)**	**0.05**	1.0
Propylparaben^a^	-2.19	(-2.57,-1.81)	< 0.001	-0.26	(-0.64,0.12)	0.2	1.0
Ethylparaben^a^	-1.09	(-1.40,-0.79)	< 0.001	-0.11	(-0.40,0.19)	0.5	0.5
Benzophenone-3^a^	1.52	(-1.94,-1.11)	< 0.001	-0.00	(-0.39,0.40)	1.0	0.8
Bisphenol S^a^	-0.05	(-0.18,0.08)	0.4	0.03	(-0.11,0.17)	0.6	0.6
Bisphenol F^a^	-0.13	(-0.34,0.07)	0.2	0.01	(-0.21,0.23)	0.9	0.2
Bisphenol A^a^	0.15	(0.0, 0.30)	0.05	-0.06	(-0.21,0.08)	0.4	0.01
2,4- DCP^a^	-0.05	(-0.19,0.08)	0.4	-0.05	(-0.18,0.09)	0.5	0.9
2,5 –DCP^b^	1.76	(1.14, 2.69)	0.01	0.77	(0.50,1.18)	0.2	0.1
Triclosan^b^	0.66	(0.40,1.06)	0.08	0.91	(0.57, 1.45)	0.7	0.5
Triclocarban^2^	0.57	(0.23,1.42)	0.2	1.40	(0.52,3.82)	0.7	0.8

^a^Multiple linear or ^b^logistic regression including sex and rhinitis adjusted for BMI, IgE, age, smoking, and time of day of urine sampling; ^c^ Interaction term between sex and current rhinitits

**Table 6:** Associations between urine biomarkers of chemical exposure and current asthma in adult participants

	**Sex: male vs female**					**Current asthma**				**Interaction**^**c**^
	**∆/OR**	**CI**		**p**		**∆/OR**		**CI**	**p**	**p**
Sum of paraben^a^	-0.68		(-0.83,-0.53)		< 0.001		-0.14	(-0.46,0.18)	0.4	0.03
Methylparaben^a^	-1.71		(-2.05,-1.38)		< 0.001		-0.05	(-0.65,0.74)	0.9	0.03
Butylparaben^b^	0.13		(0.08,0.22)		< 0.001		0.44	(0.16,1.21)	0.1	0.8
Propylparaben^a^	-2.20		(-2.57,-1.84)		< 0.001		-0.37	(-1.24,0.50)	0.4	0.9
Ethylparaben^a^	-1.11		(-1.40,-0.82)		< 0.001		-0.11	(-0.67,0.45)	0.7	0.06
Benzophenone-3^a^	-1.53		(-1.94,-1.12)		< 0.001		-0.05	(-0.86,0.75)	0.9	0.2
Bisphenol S^a^	-0.09		(-0.22,0.04)		0.2		0.09	(-0.13,0.32)	0.4	0.01
Bisphenol F^a^	-0.12		(-0.32,0.08)		0.2		0.25	(-0.18,0.68)	0.2	0.4
Bisphenol A^a^	0.19		(0.04,0.34)		0.01		-0.03	(-0.26,0.19)	0.8	0. 5
2,4- DCP^a^	-0.06		(-0.19,0.08)		0.42		0.02	(-0.28, 0.31)	0.6	0.8
2,5 –DCP^b^	1.85		(1.21,2.83)		0.004		0.47	(0.19,1.20)	0.1	0.8
Triclosan^b^	-0.06		(-0.15,0.03)		0.2		-0.08	(-0.24,0.09)	0.2	0.1
Triclocarban^b^	0.67		(0.28,1.65)		0.4		0.65	(0.09,4.95)	0.7	1.0

^a^Multiple linear or ^b^logistic regression including sex and current eczema adjusted for BMI, IgE, age, smoking, and time of day of urine sampling; ^c^Interaction term between sex and current asthma

2,4-DCP urine and bisphenol S concentrations were higher for IgE positive participants (Table 7). The test for interaction between gender and urine biomarker levels revealed significant interactions for IgE and parabensum (p = 0.03 for interaction between sex and IgE positivity). The concentration of parabensum were higher in men (beta-coefficient 0.21 (95% CI: -0.03, 0.02)), but not in women (beta-coefficient -0.07 (95% CI: -0.28, 0.13)). Adjusting for BMI or not in the models did not change the effect estimates.

**Table 7:** Associations between urine biomarkers of chemical exposure and specific IgE^d^ in adult participants

	**Sex: male vs female**			**Specific IgE**^**d**^			**Interaction**^**c**^
	**∆/OR**	**CI**	**p**	**∆/OR**	**CI**	**p**	**p**
Sum of paraben^a^	-0.66	(-0.80,-0.51)	< 0.001	-0.08	(-0.06,0.23)	0.3	0.03
Methylparaben^a^	-1.68	(-2.02,-1.34)	< 0.001	0.22	(-0.11,0.55)	0.3	0.2
Butylparaben^b^	0.14	(0.09,0.25)	< 0.001	0.66	(0.42,1.03)	0.07	0.5
Propylparaben^a^	-2.14	(-2.51,-1.77)	< 0.001	0.07	(-0.31,0.45)	0.7	0.5
Ethylparaben^a^	-1.06	(-1.35,-0.76)	< 0.001	-0.06	(-0.36,0.23)	0.4	0.7
Benzophenone-3^a^	-1.54	(-1.94,-1.14)	< 0.001	-0.23	(-0.63,0.18)	0.3	1.0
Bisphenol S^a^	-0.09	(-0.22,0.04)	0.2	**0.18**	**(-0.04,0.32)**	**0.01**	0.3
Bisphenol F^a^	-0.13	(-0.33,0.06)	1.3	-0.02	(-0.22,0.18)	0.8	0.1
Bisphenol A^a^	0.19	(0.04,0.34)	0.01	0.11	(-0.04,0.26)	0.1	0.4
2,4- DCP^a^	-0.06	(-0.19,0.08)	0.4	**0.15**	**(-0.01,0.29)**	**0.03**	0.6
2,5 –DCP^b^	1.89	(1.24 2.88)	0.03	0.84	(0.57,1.24)	0.4	0.9
Triclosan^b^	0.74	(0.46,1.19)	0.2	1.05	(0.67,1.63)	0.8	0.06
Triclocarban^b^	0.68	(0.27,1.70)	0.4	1.06	(0.39,2.10)	0.4	0.3

^a^Multiple linear or ^b^logistic regression including sex and variable for IgE, adjusted for BMI, age, smoking, and time of day of urine sampling. ^c^ Interaction term between sex and IgE. ^d^IgE positivity defined by IgE ≥ 0.35 kU/L to at least 1 of 5 allergens tested (cat, timothy grass, Cladosporium, birch, and house dust)

The estimates for associations between urine concentrations of chemicals and symptoms and disease outcomes in the adolescents are vulnerable due to the low number of girls (*n* = 44) and boys (*n* = 46), and thus, the results are not included in the tables.

Urine biomarker concentration of MPB and EPB decreased with increasing BMI (Table 8). Furthermore, statistically significant inverse associations were observed for BMI measures and 2,4-DCP and TCS urinary concentrations, and there were no statistical interaction by gender.

**Table 8:** Associations between urine biomarkers of chemical exposure and BMI in adult participants. Linear regression estimate (∆ and 95% confidence interval (CI)) for urine biomarkers applied as continuous variables, and logistic regression (OR and CI) for biomarkers applied as dichotomous variables (butylparaben, 2,5-DCP, triclosan and triclocarban)

	**Sex: male vs female**			**BMI**			**Interaction**^**c**^		
	**∆/OR**	**CI**	**p**	**∆/OR**	**CI**	**p**	**∆/OR**	**CI**	**p**
Sum of parabens^a^	-0.65	(-0.80,-0.51)	< 0.001	-0.01	(-0.03,0.01)	0.3	0.01	(-0.02,0.05)	0.4
Methylparaben^a^	-1.68	(-2.01,-1.35)	< 0.001	**-0.02**	**(-0.08,-0.00)**	**0.04**	0.04	(-0.03,0.11)	0.3
Butylparaben^b^	0.14	(0.09,0.22)	< 0.001	1.01	(0.97,1.06)	0.7	1.02	(0.92,1.12)	0.7
Propylparaben^a^	-2.16	(-2.52,-1.81)	< 0.001	-0.02	(-0.05,0.02)	0.4	0.03	(-0.04,0.10)	0.4
Ethylparaben^a^	-1.09	(-1.36,-0.81)	< 0.001	**-0.05**	**(-0.08,-0.01)**	**0.007**	0.04	(-0.03,0.10)	0.3
Benzophenone-3^a^	-1.58	(-1.97,-1.19)	< 0.001	0.02	(-0.03,0.06)	0.5	-0.07	(-0.15,0.01)	0.1
Bisphenol S^a^	-0.07	(-0.19,0.06)	0.3	0.01	(-0.01,0.02)	0.4	0.03	(-0.00,0.06)	0.07
Bisphenol F^a^	-0.14	(-0.33,0.05)	0.15	-0.00	(0.02,0.02)	0.9	-0.01	(-0.06,0.03)	0.6
Bisphenol A^a^	0.21	(0.06,0.35)	0.005	0.00	(-0.01,0.02)	0.8	0.01	(-0.02,0.04)	0.5
2,4-DCP^a^	-0.05	(-0.18,0.09)	0.5	**-0.02**	**(-0.03,-0.01)**	**0.007**	0.01	(-0.02,0.03)	0.7
2,5-DCP^b^	1.89	(1.25,2.87))	0.003	1.01	(0.96,1.06))	0.6	1.00	(0.91,1.10)	1.0
Triclosan^b^	0.81	(0.51,1.30)	0.4	**0.92**	**(0.87,0.97)**	**0.002**	1.05	(0.94,1.17)	0.4
Triclocarban^b^	0.70	(0.28,1.76)	0.5	1.06	(0.95,1.17)	0.3	0.87	(0.72,1.04)	0.1

^a^Multiple linear or ^b^logistic regression (for dichotomous exposure) including sex and BMI, adjusted for age, smoking, and time of day of urine sampling; ^c^Interaction term between sex and BMI

## Discussion

In the present study of relatively young Norwegian participants women had higher urinary concentrations of cosmetic chemicals and reported more frequent use of personal care products than men. Urine levels of parabens, in particular, correlated strongly with reported use of personal care products in both men and women. The adults and adolescents had similar prevalence of urine detection of most of the chemicals (except for TCC which was higher in adolescents). However, the adults had higher chemical concentrations in urine than adolescents. Urine biomarker concentrations of MPB, EPB, TCS and 2,4-DCP were inversely related to BMI and also lower in participants with current eczema (parabens) and IgE positivity (2,4-DCP and BPS).

Generally, women have higher levels of several phenols and parabens than men and report higher use of personal care products. In contrast to the parabens and TCS which are more prevalent among women in our study as in other studies, bisphenols and dichlorophenols were more commonly detected in urine from men. Bisphenols and TCC showed higher detection rates in adolescents than in adults, but lower median concentrations (bisphenols). The difference in detection frequency between women and men, and between adults and adolescents, may reflect a difference in exposure or differences in metabolism. Compared to our previous study on children in Norway with data collected during 2001–2004 [27], we found much lower levels of TCS in the present population of adults (27% detection and maximum concentration of 861 ng/mL) and adolescents (18.7% detection and maximum concentration of 451 ng/mL) as compared to 47% of urine samples with detectable levels and maximum of 3610 ng/mL in the previous study [27]. This is likely to reflect TCS being phased out from hygiene and cosmetic products in Norway. Elevated levels of TCS in urine have been associated with the use of increasing numbers of TCS containing products [40]. It has been speculated that TCS would be replaced with TCC. In the present study, TCC was detectable in only 5% of the samples for adult, but in 45% of the adolescents’ urine samples. Our results are in contrast with US populations with the highest detection frequency observed among adults [41], and thus indicating geographic differences in regulation and use of cosmetic chemicals. Dermal exposure from personal care products is believed to be the main route of human exposure to TCC [41], and the divergence in concentration of TCC observed between adult and adolescents in the present study may be the result of difference in pattern of use, however, the low prevalence of levels above LOD does not allow firm conclusions.

Contrary to TCS and TCC, we found that the detection frequency of PPB, MPB and BPA were similar for adults and adolescents (94–99%), as was also the case for BPB (> 86%). However, common for all chemicals were higher concentrations observed for adults compared to adolescents. Further, we found a higher concentration of MPB than for PPB, but with a strong correlation between them, probably explained by the fact that MPB and PPB often are used in combination in the products [6, 42]. A study from Denmark, assessing paraben content in cosmetic products, showed that nearly all leave-on products contained parabens [43]. The most common parabens were methyl, followed by ethyl > propyl > butyl > benzyl paraben. New EU regulations from 2014 (applied in Norway from 2015) state that the sum of PPB and BPB in cosmetic products cannot exceed 0.14% (0.19% as esters), and mixtures of PPB, BPB, MPB, EPB are not to exceed 0.8% [44]. Thus, the industry has, to some extent, been forced to replace parabens with other preservatives, which might have influenced the relatively low level of BPB reported in the present study. BPF and BPS are increasingly replacing BPA in polycarbonates manufacturing. Bisphenols may be found in personal care products, and although dermal exposure and dermal absorption is expected to be low [13], it should not be ignored [14].

We found a strikingly high correlation between frequency of use of personal care products and urinary levels of parabens, especially for leave-on products. Both MPB and PPB are permitted for use as direct food additives and as indirect food additives in food packaging materials [6, 42]. However, it has been indicated that only 0.6–0.8% of the total paraben exposure are dietary exposure and in a previous study no association between concentration of parabens in food and packing materials was found [45]. Other sources, such as indoor dust, contribute less than 1% to the body burden of exposure [46]. Thus, it is believed that the main contributing sources for humans are personal care products [7]. Previous studies have also found that sex, age, race/ethnicity and household income have been associated with urine concentration of parabens, and this is in line with characteristics associated with other compounds where personal care products are the main source of exposure, e.g. DEP [47] and BP3 [10]. In the NHANES study, elevated levels of parabens and BP3 were associated with daily use of mouthwash and sunscreen [3]. Thus, while there are several sources of exposure, for level of parabens we and others find that personal care products are of particular importance [48].

In our study, concentration of parabensum, propylparaben, methylparaben and butylparaben were lower in adult with current eczema compared to those without current eczema. This is contrary to expectations, as increased use of ointment due to skin conditions would be expected to lead to higher levels of parabens and other chemicals commonly found in personal care products, as reported for children with atopic dermatitis who frequently use emollients [49]. We speculate that this could be because adults with current eczema may have increased awareness when choosing a product and more commonly choose paraben free products. Current eczema did not differ significantly by BMI-categories. BPA concentration was decreased with current rhinitis for men. BPS increased with current asthma in women, and a positive association was seen between increasing BPS and specific IgE alone. BPS is a substitution for BPA, with similar chemical structure, however, data on toxicity is limited [50]. Mendy et al. reported that exposure to BPF and BPS is associated with asthma and/or hay fever [51]. For 2,4-DCP we observed a positive association for specific IgE. Savage et al. [26] found that parabens and TCS were associated with aeroallergen and food allergen sensitization, and that boys mainly drove the association with TCS. Other studies have also found higher odds of environmental sensitization in boys with increasing biomarkers of parabens [52]. Earlier, we have reported TCS concentration to be associated with allergic sensitization and rhinitis in children [27]. However, in the present study the low levels of TCS and TCC constitutes relatively “rare” exposures with lower statistical power to detect associations with outcomes, which may explain why we did not observe similar associations for TCS in the present study.

Urine biomarker concentration of MPB and EPB, TCS and 2,4-DCP decreased with increasing BMI for both genders. MPB and EPB also showed the highest maximum concentrations in urine and increased with increasing frequency of use of several personal care products. However, the frequency of use of personal care products did not differ by BMI-categories. Recently, parabens and BP3 were found to be stored in adipose tissue [21]. Thus, given that these phenolic compounds are detectable in many body fluids as well as have the potential to be stored in adipose tissue, shows that these compounds are distributed throughout the body, regardless of the route of exposure [17, 18]. Sequestration of parabens will be greater with greater body fat, thus a person with higher fat stores may excrete lower amount of paraben metabolites in urine. Parabens and BP3 have been reported to show hormonal activity [53] and adipose tissue is viewed as an endocrine organ, but whether paraben accumulation in fat affect metabolic processes is not known. Hu et al. suggested that parabens may play a role in development of obesity [54] and inverse associations between paraben exposure and adiposity markers has been reported recently, with more pronounced associations among females [55]. One other study found concentrations of MPB and PPB to be lower among obese participants [56]. Neither of these two studies had collected information about use of personal care products. The correlation coefficient was low for MPB and TCS (r = 0.17) and for MPB and 2,4-DCP (r = 0.21) in our study indicating that these associations were independent of exposure to other phenolic compounds. Severe obesity is shown to have a potential dilution effect on lipophilic chemicals, whose concentration may decrease with increased volume of adipose tissue [57]. Our result for MPB, the chemical with highest measured urine concentration, could support this explanation; a high amount of the chemicals leads to saturation of storage in adipose tissue, that are not observed for chemicals to which we are less exposed.

The strengths of this study are the well-defined study-population with state-of-the-art measurements of urine chemicals, and in addition information on personal care products. Non-persistent phenolic compounds like parabens and BP3 are excreted from the body mainly through urine. For parabens, the concentration of total (free and conjugated) urine species of the parent parabens can be used as valid human exposure biomarkers [19]. Studies that have compared the concentration of parabens, BP3 and TCS in different matrices, found that urine was a much better matrix than serum (e.g. only 30% detection of BP3 in serum compared to 100% detection in urine) [17]. In the National Health and Nutrition Examination Survey (NHANES) it was shown that persons in high household income categories had higher urinary concentration of parabens (MPB and PPB) than persons in medium or low household income categories [4]. We have discussed if education should be included, however, the population is young, and analysis also show that it will not affect the results. In the present study, smoking was not the main outcome, and thus, we did not prioritize to measure cotinine levels. We do not find it likely that our study participants under- or overreport smoking, and in previous publications from Norway, self-reported tobacco use was found to correspond well to cotinine levels in plasma [58]. All the participants in this study first completed a self-reported questionnaire and was later interviewed by study nurses during the clinical examination. The questionnaires and interviews have been used in several population-based multicenter studies (www.rhine.nu and www.ecrhs.org) and for many phases of follow-up for those study participants. The definitions of current eczema, asthma and rhinitis based on these interview and questionnaires have been applied in multiple studies [34–36] and thus these outcome definitions were applied in order to be consistent with previous publications. Previous studies have also found that “Do you have or have you ever had asthma” provide prevalence estimates comparable to clinical diagnosis [59]. We cannot confirm a specific diagnosis of atopic dermatitis, but our definition of current eczema is readily used in epidemiological studies [60]. Further, we cannot rule out the possibility of misclassification in the outcome, but it is highly unlikely that such misclassification could be related to measured urinary levels of chemicals or reported use of personal products. Thus, it is likely that misclassification would be non-differential and reduce the observed associations towards the null, but not create spurious results.

The questionnaire that was used to obtain information about frequency of use of personal care products in this study was detailed. The questionnaire has not been validated, but was developed through an expert group discussion for the use in the Respiratory Health in Northern Europe (RHINE) survey and has since been used in the last follow-up of the European Community Respiratory Health Survey (ECRHS) [61]. An exposure-mixture approach was discussed for the study. A limitation by not to follow a multi-pollutant approach is the risk of losing synergistic effects. However, because most of the chemicals derive from different sources and are not known to exert similar effects on health outcomes, we limited the approach to parabens. Often more than one paraben is used as additives in personal care products, and thus, the urinary concentrations of the parabens are combined into molar sum of all four parabens.

The present study is the first to report results from the personal care product questionnaire. The urinary concentrations of parabens and BP3 increased in a dose–response manner with frequency of use of personal care products, allowing for identification of a positive association between the use of cosmetics products and the levels of cosmetic chemical biomarkers in urine. However, no analytical measurement of chemicals was done on the products themselves and we did not obtain information on the cosmetic brands that had been used by the participants. Urine was collected as a spot sample, but time of day of urine collection was recorded and specific gravity was used to account for urine dilution. Although the phenolic compounds have relatively short half-life, the daily use of personal care products and often continued use of the same brands, lead to continuous exposure to the compounds [62, 63]. Thus, we can assume that the exposures to these chemicals are more or less consistent over a prolonged period of time [52]. Fisher et al. found that a single spot urine sample was representative of a person’s daily exposure to parabens [48], and Hines et al. found a significant correlation between the first and second visit for seven out of nine phenols, suggesting consistent and/or recurrent exposure to these compounds [17]. Although Hines et al. did not find a good correlation for BP3, others have demonstrated fair to good reliability for one urine sample for estimating exposure to phenol metabolites like BP3 and TCS [62, 64, 65]. BP3 is regulated as UV filters and is a common component in topical sunscreen, but also in other personal care products, such as moisturizers, shampoos, hair care products, cosmetics, nail polish, plastic materials and dental composite materials. The major human exposure pathway for BP3 is dermal absorption from the use of consumer products [18]. The limitations is the cross-sectional design of this study which does not allow the inference of causal relationships.

## Conclusions

Urine measurements showed high body burden of exposure to antibacterial and cosmetic chemicals commonly used in personal care products. The majority of the chemicals were present in both adults and adolescent with similar prevalence, but for most of the chemicals the adults had higher concentrations than adolescents, possibly due to higher use of personal care products in adults. For most products, women reported more frequent use than men, and the use of personal care products strongly correlated with urine measurements. Our findings suggest an inverse association between urine biomarker concentrations of the cosmetic phenols and BMI, which may be due to fat affinity of the chemicals. Interestingly, lower levels of parabens were observed for participants with current eczema. Our results highlight potential impact of chemical exposures on human health, with an urgent need for better understanding of these very widespread exposures specifically over time as in longitudinal studies.

## Supplementary Information

**Additional file 1.**

**Additional file 2.**

**Additional file 3.**

## Data Availability

The dataset used and analysed during the current study is available upon request.

## Abbreviations

BMI: Body mass index
CI: Confidence interval
LOD: Limit of detection
BPA: Bisphenol A
BPF: Bisphenol F
BPS: Bisphenol S
TCS: Triclosan
TCC: Triclocarban
PPB: Propylparaben
MPB: Methyl paraben
2,5-DCP: 2,5 Diclorophenols
2,4-DCP: 2,4 Diclorophenols
BP3: Benzophenone-3
SG: Urine specific gravity
IgE: Immunoglobulin E
