# Supplementary material for: Exposure to environmental phenols and parabens, and relation to body mass index, eczema and respiratory outcomes in the Norwegian RHINESSA study
Source: Environ Health. 2021 Jul 13;20:81. doi: 10.1186/s12940-021-00767-2 (PMC8278607; doi:10.1186/s12940-021-00767-2)

Supplemental Figure S1: Adjusted linear predicton of urine concentration of TCC, parabensum (sum of all parabens) and MPB by age groups, illustrated separately for females (left column graphs) and males (right column graphs)

a) TCC by age (females)

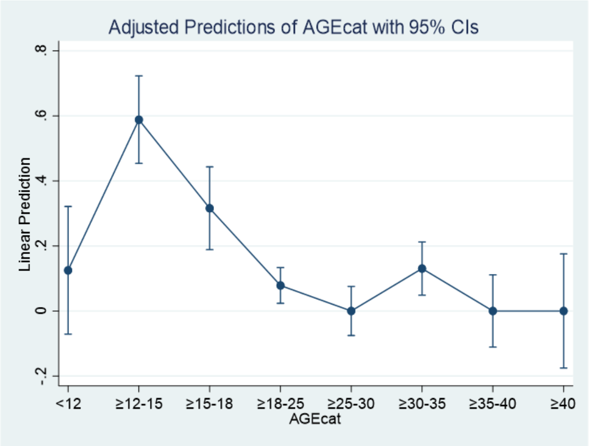


b) TCC by age (males)

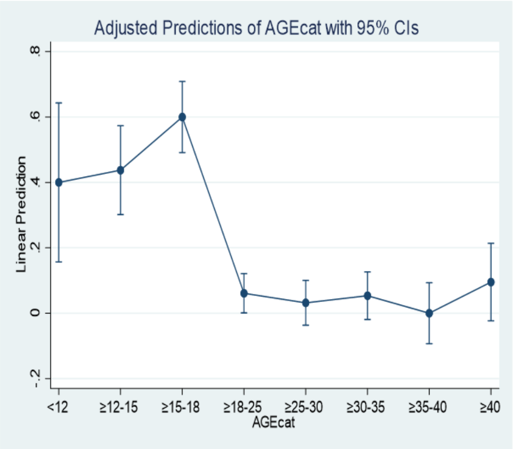


c) Parabensum by age (females)

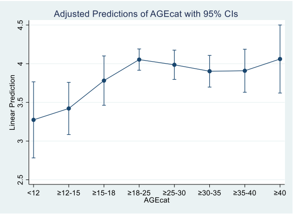


d) Parabensum by age (males)

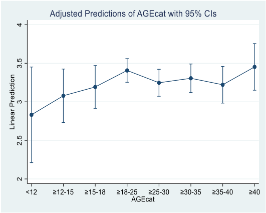


e) Methylparaben by age (females)

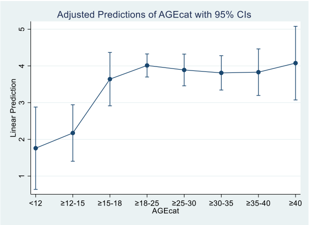


f) Methylparaben by age (males)

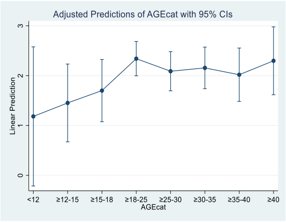

Supplement: Supplementary file 1 — Additional file 1. [file 12940_2021_767_MOESM1_ESM.pdf]
