# Supplementary material for: Exposure to environmental phenols and parabens, and relation to body mass index, eczema and respiratory outcomes in the Norwegian RHINESSA study
Source: Environ Health. 2021 Jul 13;20:81. doi: 10.1186/s12940-021-00767-2 (PMC8278607; doi:10.1186/s12940-021-00767-2)
Supplement: Supplementary file 2 — Additional file 2. [file 12940_2021_767_MOESM2_ESM.pdf]

Supplemental Table S1: Distribution of SG-corrected urinary phenols and triclocarban concentrations (ng/mL) among 496 RHINESSA adults, women and men

| **Analyte (Long name)** | Analyte | LOD | group | % ≥ LOD | 5th | 25th | 50th | 75th | 95th | Max |
|---|---|---|---|---|---|---|---|---|---|---|
| **Methylparaben** | MPB | 1.0 | Female | 98.7 | 3.13 | 17.4 | 60.5 | 172 | 619 | 3727 |
| | | | Male | 91.8 | <LOD | 2.61 | 6.79 | 22.5 | 249 | 1355 |
| **Ethylparaben** | EPB | 1.0 | Female | 73.5 | <LOD | 1.04 | 3.28 | 13.7 | 83.6 | 2957 |
| | | | Male | 51.6 | <LOD | <LOD | <LOD | 2.48 | 12.0 | 707 |
| **Propylparaben** | PPB | 0.1 | Female | 99.6 | 0.30 | 1.88 | 7.58 | 32.5 | 158.6 | 781 |
| | | | Male | 91.1 | <LOD | 0.26 | 0.55 | 1.72 | 39.2 | 460 |
| **Butylparaben** | BPB | 0.1 | Female | 60.9 | <LOD | 0.10 | 0.22 | 0.97 | 13.1 | 137 |
| | | | Male | 17.0 | <LOD | <LOD | <LOD | 0.14 | 2.06 | 23.1 |
| **Bisphenol A** | BPA | 0.2 | Female | 92.8 | 0.39 | 0.78 | 1.21 | 2.05 | 5.22 | 16.7 |
| | | | Male | 97.7 | 0.45 | 0.95 | 1.57 | 2.67 | 5.03 | 22.0 |
| **Bisphenol F** | BPF | 0.2 | Female | 49.6 | <LOD | <LOD | 0.31 | 0.49 | 1.63 | 80.6 |
| | | | Male | 50.4 | <LOD | <LOD | 0.24 | 0.50 | 2.96 | 20.5 |
| **Bisphenol S** | BPS | 0.1 | Female | 58.4 | <LOD | 0.12 | 0.15 | 0.26 | 0.44 | 3.13 |
| | | | Male | 65.1 | <LOD | <LOD | 0.14 | 0.22 | 0.63 | 8.36 |
| **Benzophenone-3** | BP-3 | 0.4 | Female | 90.3 | 0.55 | 5.49 | 17.6 | 73.9 | 1470 | 6041 |
| | | | Male | 82.4 | <LOD | 1.53 | 4.24 | 12.5 | 94.6 | 2075 |
| **2, 5-diclorophenol** | 2,5-DCP | 0.1 | Female | 30.3 | <LOD | <LOD | 0.12 | 0.19 | 0.55 | 4.13 |
| | | | Male | 45.3 | <LOD | <LOD | 0.11 | 0.18 | 0.62 | 23.5 |
| **2, 4-diclorophenol** | 2,4-DCP | 0.1 | Female | 58.8 | <LOD | 0.12 | 0.17 | 0.23 | 0.52 | 3.13 |
| | | | Male | 71.7 | <LOD | 0.10 | 0.15 | 0.21 | 0.78 | 7.45 |
| **Triclosan** | TCS | 1.7 | Female | 30.7 | <LOD | <LOD | 1.45 | 2.82 | 39.2 | 861 |
| | | | Male | 23.3 | <LOD | <LOD | <LOD | 2.02 | 69.1 | 513 |
| **Triclocarban** | TCC | 0.1 | Female | 4.7 | <LOD | <LOD | <LOD | 0.14 | 0.31 | 0.94 |
| | | | Male | 4.7 | <LOD | <LOD | <LOD | 0.10 | 0.18 | 0.55 |

LOD: Limit of detection
