# Supplementary material for: Exposure to environmental phenols and parabens, and relation to body mass index, eczema and respiratory outcomes in the Norwegian RHINESSA study
Source: Environ Health. 2021 Jul 13;20:81. doi: 10.1186/s12940-021-00767-2 (PMC8278607; doi:10.1186/s12940-021-00767-2)
Supplement: Supplementary file 3 — Additional file 3. [file 12940_2021_767_MOESM3_ESM.pdf]

Supplemental Table S2: Urine concentrations* (ng/mL) of methylparaben (specific gravity adjusted) by reported frequency of use of personal care products among 496 RHINESSA adults, women and men

		Reported frequency of use			
Personal care products**		Never or <1/week	1-3 days/week	4-7 days/week or > once daily	p-value***
Perfume spray	Female	22 (2.4-320) [3726]	68 (5.5-338) [1033]	68 (5.9-347) [3269]	**0.03**
	Male	5 (1.3-64) [1355]	5 (1.0 -177) [549]	11 (2.1-118) [957]	**0.002**
Perfume	Female	61 (5.0-338) [3270]	17 (8.9-51) [51]	108(2.4-267)[3726]	0.2
Deostick	Female	33 (20.9-232) [232]	15 (4.0-386) [386]	66 (4.9 -336) [3727]	0.2
	Male	4 (1.8-38) [101]	5 (1.4-15.4) [17]	7 (1.4-110) [1355]	0.3
Deo-spray	Male	7 (1.4-104) [1355]	4 (1.0-22.6) [39]	7 (1.8-42) [101]	0.4
Moisturizing cream	Female	13 (2.2-69) [165]	19 (3.4-188) [683]	87 (8.2-386) [3727]	**0.0001**
	Male	5 (1.3-38) [957]	7 (1.8-63) [316]	24 (2.6-381) [1355]	**0.0001**
Lotions	Female	22 (2.4-166) [749]	83 (4.9-351) [3270]	89 (11.8-342) [3727]	**0.0001**
Cleansing cream	Female	25 (3.4-336) [3726)]	49 (4-355) [614]	109 (9.6-325) [3270]	**0.0002**
Make-up	Female	21 (2.1-749) [3726]	49 (3.4-245) [337]	67 (6.0-325) [3270]	0.08
Nail-care	Female	46 (4.1-314) [3726)]	88 (9.6-639) [3270]	77 (6.3-232) [550]	0.08
Hairspray	Female	49 (4-297) [3726]	75 (12.4- 587) [1824]	143 (10.6-386) [3270]	0.06
Hair-styling products	Female	43 (4.0-338) [3726]	72 (12.7-233) [3270]	93 (6.3-386) [639]	0.1
	Male	5 (1.1-177) [786]	4 (1.6-14.3) [125]	16 (1.5 -87) [1148]	**0.08**
Shaving-products	Male	7 (1.2-358) [1148]	5 (1.4-42) [786]	14 (2.8-387) [387]	0.3
After-shave	Male	6 (1.3-316) [1148]	4(1.0 -102) [125]	23 (2.8-43) [43]	0.5
Hair-color†	Female	56 (3.7-386) [3726]	39 (6.0-268) [1033]	161 (12.7-386) [801]	**0.008**
Hair-bleach†	Female	60 (3.7-386) [3726]	43 (8.2-280) [3270]	107 (10.8-232) [232]	0.9

*Median (10th percentile – 90ieth percentile) [max].**Due to large gender differences in reported use for some products, use of deospray, shaving products and after-shave are only shown for men, and lotions, perfume, cleansing cream, make-up, nail-care, hairspray, hair-color and hair-bleach are only shown for women.***P-value from Kruskal-Wallis for differences between urine concentrations by frequency of use of personal care products
